# Surveillance for rhabdomyolysis after the consumption of crayfish in Wuhan, China, 2016–2022

**DOI:** 10.3389/fnut.2024.1333888

**Published:** 2024-05-03

**Authors:** Yating Wu, Xiao Wang, Xiaoye Wang, Zhenyu He, Rui Wang, Zhihan Chen, Xiaomin Wu

**Affiliations:** ^1^Institution of Food and Environmental Hygiene, Wuhan Center for Disease Control and Prevention, Wuhan, China; ^2^Public Health Emergency Center, Chinese Center for Disease Control and Prevention, Beijing, China; ^3^College of Forestry and Landscape Architecture, Guangzhou South China Agricultural University, Guangzhou, China

**Keywords:** rhabdomyolysis, Haff disease, crayfish, outbreak, Wuhan

## Abstract

**Objectives:**

To analyze the epidemiological characteristics and etiology of crayfish-related rhabdomyolysis.

**Methods:**

Cases of crayfish-related rhabdomyolysis in Wuhan were monitored, and professional training of city’s surveillance personnel was conducted. Unified questionnaires were used to collect data.

**Results:**

The first case of crayfish-related rhabdomyolysis occurred on July 12, 2016. Subsequently, 423 patients were reported over the next 7 years, with muscle pain, weakness, and chest distress as main symptoms. In total, 64.54% (273/423) of patients were females, and young adults (aged 20–49 years) account for 86.22% (363/423) of patients. The primary clinical presentations were muscle pain, muscle weakness, and chest discomfort. The median incubation time was 6 h. And the number of cases may be related to water levels in Yangzi river. Laboratory tests revealed elevated creatine kinase and myoglobin levels. In total, 95.16% (236/248) of patients had consumed crayfish tail shrimp and 91.53% (227/248) had consumed crayfish liver and pancreas (Female crayfish also contain ovaries). Only 25.00% (62/248) of patients had a history of alcohol consumption. On average, 227 patients consumed 15 (3–50) crayfish, of whom 84.14% (191/227) consumed more than 10 crayfish. All patients had a favorable prognosis.

**Conclusion:**

Crayfish-related rhabdomyolysis is a kind of a case or cluster of patients present with severe myalgia or weakness of unknown etiology and mechanism disease in Wuhan, China, 2016–2022. Excessive consumption of crayfish may be a risk factor for the disease. The relationship between the specific parts of crayfish consumed and the onset of the disease is unclear, suggesting further research is needed to identify the relevant risk factors for the disease.

## 1 Introduction

Rhabdomyolysis (RM) is a group of clinical syndromes characterized by muscle pain, muscle weakness, and brown-colored urine, as well a tissue and organ damage. Due to the disintegration of cell contents and the release of large amounts of myoglobin, creatine kinase (CK), and lactate dehydrogenase into the peripheral blood (1), laboratory tests showed increased plasma levels of CK and myoglobin.

Haff disease, which patients had unexplained rhabdomyolysis within 24 h after eating aquatic products, was initially described in Europe in 1924 (2). Since then, various countries, including Australia (3), the US (4), and Brazil (5, 6), have reported cases of Haff disease caused by the consumption of a variety of fresh water and sea water products (7, 8). In China, Haff disease first occurred in Guangdong in 2009 and Beijing in 2000 (9). Subsequent outbreaks mostly occurred along Yangtze River, from Nanjing, Yangzhou, Huai’an, to Yancheng, all in Jiangsu Province. Isolated outbreaks occurred in other cities since 2010—Shijiazhuang, Baoding in Hebei Province, Yueyang in Hunan Province, Shanghai, Wuhu in Anhui Province, Shenzhen in Guangdong Province and Hong Kong (imported cases from Shenzhen) (9, 10).

Since 2016, a large quantity of crayfish-related RM happened along Yangtze River (11, 12), crayfish-related RM has been reported in Nanjing, Jiangsu Province (13, 14), Tonglin (11), Wuhu (15), Ma’anshan (16) in Anhui Province, Shenzhen (17), Guangzhou (18) in Guangdong Province, and other areas in China (19). Outbreaks occurred predominately in the summer. Crayfish accounted for almost all the outbreaks.

A previous outbreak of crayfish caused RM in China was reported in Anhui in 2016 (11). We identified that the outbreak in Wuhan had a similar pattern to the Anhui outbreak. The epidemic curve was characterized by persistent point source exposure. The peak of onset was between the end of July and the beginning of August, and the peak platform lasted for 8 days, which was almost 2 weeks earlier than the suspected foodborne RM outbreak in Anhui and Jiangsu in 2016 (11, 20). The duration of the peak platform was significantly reduced.

In the summer of 2016, there was an unexplained case of RM related to crayfish consumption in southern Anhui (11). Since the first suspected case of foodborne RM syndrome was reported from Wuhan in 2010, the Wuhan Center for Disease Control and Prevention has been carefully monitoring the incidence of RM. Subsequently, there have been similar outbreaks in Wuhan, Hubei province, between July and August, 2017. In total, 183 cases of RM caused by crayfish consumption were reported between July 17 and September 10.

This study is based on the outbreaks of Yangtze river crayfish-related RM using data obtained from the Chinese Center for Disease Prevention and Control. We administered a questionnaire to collect the basic information, clinical manifestations, laboratory test results, crayfish consumption habits, and epidemiological characteristics related to Wuhan crayfish-related RM.

## 2 Materials and methods

The incidence data of foodborne RM syndrome in Wuhan between 2016 and 2020 were obtained from the Foodborne Disease Case Reporting Information System of the National Center for Food Safety Risk Assessment, which encompasses 222 medical institutions that cover the entire city of Wuhan. The reporting personnel in the medical institutions were trained systematically. The cases of RM were diagnosed clinically. The demographic data are from the statistical yearbook of Wuhan Bureau of Statistics.

Descriptive epidemiological methods were utilized to analyze the characteristics of foodborne RM by time of diagnosis, region, and population in Wuhan. The relationship between the number of cases and water levels in Wuhan Yangzi river is statistically analyzed using linear correlation. Excel 2010 and SPSS 13.0 software were used for data processing and analysis. The maps were constructed using the ArcGIS 10.2 software.

### 2.1 Case definition

The cases included patients who fulfilled the required epidemiological, clinical, and laboratory criteria in Wuhan, China, 2016–2022. The epidemiological criteria included a history of consumption of crayfish (crayfish and mantis clams) and other aquatic products within 24 h in 2016–2022, excluding patients with excessive exercise, trauma, alcohol consumption, autoimmune diseases, genetic diseases, drugs, infections, or metabolic abnormalities. The clinical criteria included systemic or local muscle pain accompanied by fatigue and other symptoms of RM following consumption of crayfish and other aquatic products within 24 h. The laboratory parameters included (a) elevated serum or urinary myoglobin levels; (b) serum CK level between the reference value and 5 times higher than the reference value; and (c) serum CK level 5 times or more than the reference value of serum CK level.

Suspected cases included those who fulfilled the epidemiological, clinical, and laboratory criteria (b). Furthermore, probable cases included those who fulfilled the epidemiological, clinical, and laboratory criteria (a). Confirmed cases included those who fulfilled the epidemiological, clinical, and laboratory criteria (a) and/or (c). All the case definition comes from the Manual of Special Monitoring on Rhabdomyolysis Syndrome of the Chinese Center for Disease Control and Prevention.

## 3 Results

### 3.1 Outbreak description

**Figure 1:**
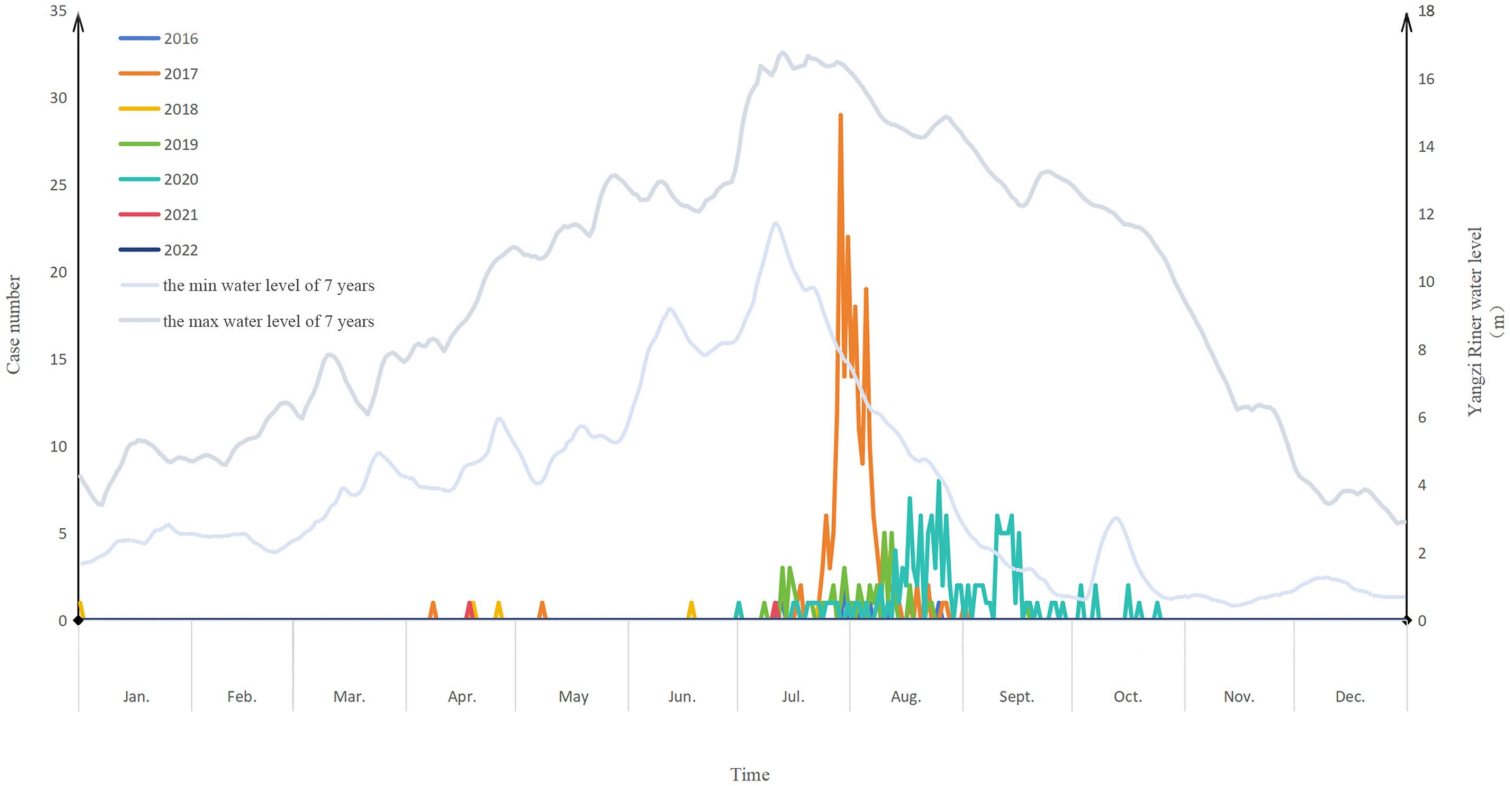
The number of rhabdomyolysis cases after the consumption of crayfish and the water level of Yangzi river in Wuhan, China during 2016–2022.

A total of 423 cases were reported from Wuhan between 2016 and 2022, including 14 cases in 2016, 213 cases in 2017, 49 cases in 2019, 5 cases in 2018, 140 cases in 2020, 2 cases in 2021 and 0 in 2022 (Supplementary material). The morbidity was 0.13/100,000 in 2016, 1.98/100,000 in 2017, 0.05/100,000 in 2018, 0.44/100,000 in 2019, 1.25/100,000 in 2020, 0.02/100,000 in 2021 and 0 in 2022 (Supplementary material). The morbidity rates were significantly different between years (*X*^2^ = 759.19, *p*<0.001). The cases included 240 suspected cases, 17 probable cases, and 166 clinically confirmed cases. The number of cases increased with the rising water level in Wuhan Yangzi river. In the years with a high incidence rate, the correlation between the number of cases and the water level was stronger, as evidenced by data from 2017 (*R* = 0.32, *p* < 0.01), 2019 (*R* = 0.40, *p* < 0.01), and especially 2020 (*R* = 0.53, *p* < 0.01) (Figure 1).

Most cases were reported between July 13 and October 19 each year (Figure 1). Once local health authorities began active surveillance for cases, the outpost hospitals reported these cases. During 2016–2022, the first case appeared on July 12, 2016, whereas the last case occurred on July 11, 2022. The number of cases was significantly higher in 2017 (213 cases) and 2020 (140 cases) compared to the other years. However, unlike 2020, the other years exhibited a scattered distribution of cases. In the year 2017, the cases occurred within a few days, reaching the peak on July 29. The shortest incubation duration among the 423 investigated cases was 0 h, whereas the longest was 22 h, with a median incubation duration of 6 h.

**Figure 2:**
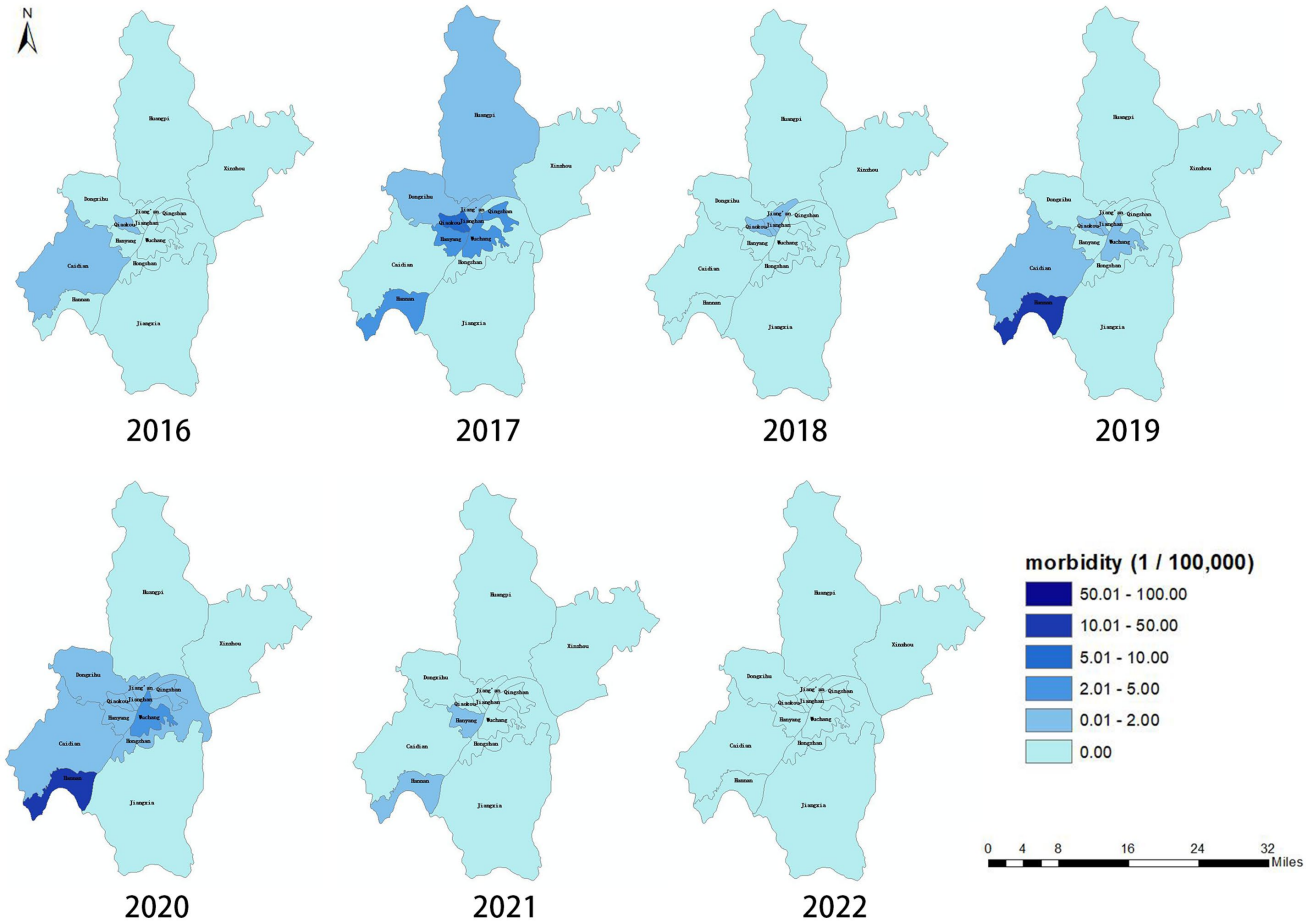
Regional distribution of outbreak of Rhabdomyolysis after the consumption of crayfish in Wuhan, China, 2016–2022 (*n* = 423).

There are 13 administrative regions in Wuhan, 11 of which have reported cases (except for Jiangxia and Xinzhou regions). The regions with the highest morbidity were Hannan, Jianghan, Jang’an, and Wuchang districts, in this order (Figure 2).

**Figure 3:**
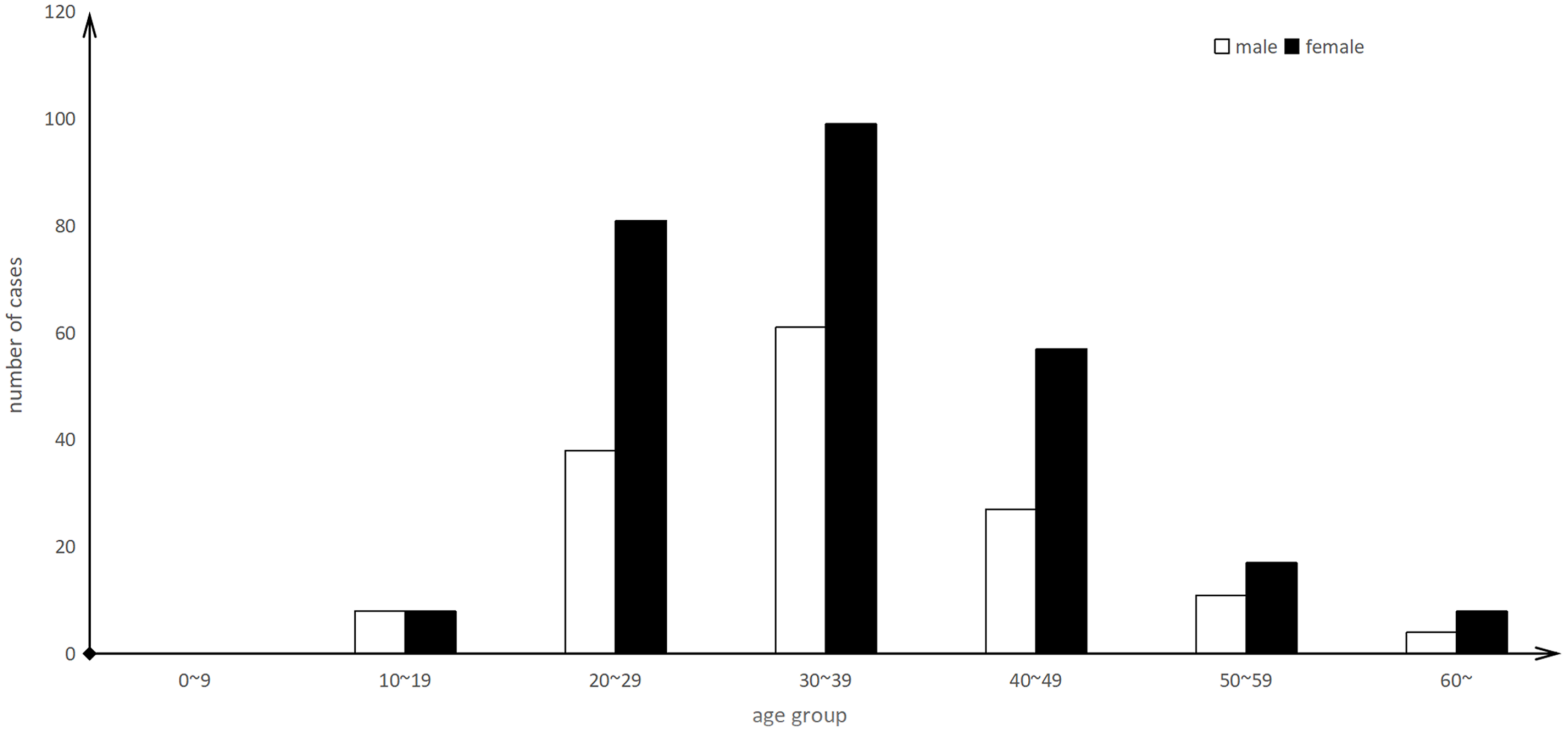
Age distribution of outbreak of Rhabdomyolysis after the consumption of crayfish in Wuhan, China, 2016–2022 (*n* = 248).

Female patients accounted for 64.54% (273/423) of cases. The age of patients ranged from 12 to 84 years, and young adults (aged 20–49 years) accounted for 86.22% (363/423) of the cases (Figure 3).

Most cases reported sudden onset of muscle pain (405/423, 95.74%), particularly backache (245/423, 57.92%), followed by weakness (244/423, 57.68%), and chest discomfort (160/423, 37.83%) (Table 1). There were no fatalities during this outbreak.

**Table 1:** Clinical characteristics of cases in outbreak of Rhabdomyolysis after the consumption of crayfish in Wuhan, China, 2016–2022 (*n* = 423).

Sign or symptoms		Number	Proportion (%)
Muscle pain		405	95.74
	Back	245	57.92
	Shoulder	165	39.01
	Whole body	104	24.59
	Chest	33	7.80
	Waist	176	41.61
	Neck	176	41.61
	Upper limb	115	27.19
	Lower limbs	104	24.59
Muscle weakness		244	57.68
Chest distress		160	37.83
Difficulty breathing		125	29.55
Dark urine		94	22.22
Emesis		76	17.97
Dizziness		61	14.42
Stomachache		55	13.00
Neural paralysis		53	12.53
Dry mouth		51	12.06
Diarrhea		49	11.58
Headache		32	7.57
Touch pain		26	6.15
Fever		10	2.36

### 3.2 Laboratory investigations

The following laboratory parameters were tested: myoglobin, CK, CK isozyme, aspartate aminotransferase, alanine aminotransferase, lactate dehydrogenase, urinary occult blood, proteinuria, uric acid, urea nitrogen, and creatinine. Of these, significantly increased levels were found for myoglobin (125/131, 95.42%) and CK (212/220, 96.36%) and were significantly abnormal levels were found for liver function tests (alanine aminotransferase 115/133, 86.47%) and aspartate aminotransferase (138/151, 91.39%), whereas the renal function tests were also partly altered (Table 2).

**Table 2:** The abnormal laboratory values of cases in outbreak of habdomyolysis after the consumption of crayfish, Wuhan, China, 2016–2022 (*n* = 423).

Laboratory values	Test number	Median (range)	Reference value	Abnormal number	Pro (%)
Myoglobin	131	664.5 (11.7–10705.76) μg/L	25–72 μg/L	125	95.42
Creatine kinase (CK)	220	2,551 (24–54566) U/L	0–167 U/L	212	96.36
Creatine kinase isozyme(CK-MB)	138	124.8 (0.2–7300) U /L	0–25 U/L	135	97.83
Aspartate aminotransferase (AST)	151	84 (14–1175) U/L	8–40 U/L	138	91.39
Lactic dehydrogenase (LDH)	157	258 (113–1965) U/L	103–227 U/L	144	91.72
Alanine aminotransferase (ALT)	133	84 (14–1175) U/L	5–40 U/L	115	86.47
Urinary occult blood	77	+(− ~4+)	Negative	44	57.14
Proteinuria	62	−(− ~4+)	Negative	25	40.32
Uric acid	150	285.9 (3.2–809.5) U/L	142-339 μmol/L	53	35.33
Urea nitrogen	144	4.5 (1.07–724) U/L	1.79–7.14 mmol/L	20	13.89
Creatinine	157	58.6 (33.8–360) U/L	33-133 μmol/L	1	0.64

Since clinical testing is a paid program, we use a voluntary model.

### 3.3 Epidemiological exposure and food consumption

During the interviews, patients were asked about possible exposure to other cases, food consumption, and the temporal relation with symptom onset.

All 423 patients had consumed crayfish within 24 h before symptom onset. However, only 248 cases had their food intake surveyed, of which 25.00% (62/248) had a history of alcohol consumption, 45.16% (112/248) of the cases ate crayfish with a cooking time of 15 min or more. 95.16% (236/248) had crayfish tail shrimp consumed, and 91.53% (227/248) had crayfish liver and pancreas consumed (Female crayfish also contain ovaries). Furthermore, 77.02% (191/248) of cases consumed more than 10 crayfish. Crayfish heads were not removed in 47.58% (118/248) of cases. Their gills were not clipped in 53.63% (133/248) of cases (Table 3).

**Table 3:** The composition ratio of related influencing factors in outbreak of Rhabdomyolysis after the consumption of crayfish, Wuhan, China, 2016–2020 (*n* = 248).

Influencing factors	Classify	Number	Composition ratio (%)
Consumption	<10	36	14.52
	> = 10	191	77.02
	Unkown	21	8.47
Crayfish liver and pancreas (Female crayfish also contain ovaries)	Yes	227	91.53
	No	21	8.47
Eat crayfish tail shrimp	Yes	236	95.16
	No	12	4.84
Eat crayfish intestines	Yes	9	3.63
	No	239	96.37
Source	Fishing	11	4.44
	Itinerant vendor	16	6.45
	Farmer’s market	187	75.40
	other	34	13.71
Cooking time (min)	0–14	12	4.84
	15–30	88	35.48
	>30	23	9.27
	Unkown	125	50.40
Brush cleaning	Yes	145	58.47
	No	25	10.08
	Unkown	78	31.45
Crayfish head removed	Yes	65	26.21
	No	118	47.58
	Unkown	65	26.21
Crayfish gills removed	Yes	46	18.55
	No	133	53.63
	Unkown	69	27.82
Crayfish intestines removed	Yes	137	55.24
	No	34	13.71
	Unkown	77	31.05
Cooking methods	Braise in sauce	58	23.39
	Steamed	66	26.61
	Braised in oil	60	24.19
	other	22	8.87
	Unkown	42	16.94
Drink	Yes	62	25.00
	No	186	75.00
Drink beer	Yes	51	20.56
	No	197	79.44

## 4 Discussion

In China, the crayfish that people eat are the *Procambarus clarkii*. Native to northern Mexico and southern United States, crayfish are now widely distributed in more than 40 countries and regions, and are also widely present in all parts of China, especially in the middle and lower reaches of the Yangzi River. Crayfish are found in rivers, lakes, ditches, ponds and rice fields. It lives in shallow wetlands, lakes and water with abundant grasses.

The periodicity of crayfish consumption is consistent with the production of crayfish, with the off-season from January to February and September to December, and the consumption shows an explosive growth from March, reaching a peak in May to June, and then starting to decline. The peak season for crayfish consumption is from April to August each year (21).

Chinese cuisine offers a diverse range of ways to prepare crayfish, but two standout methods are particularly popular: steamed crayfish and spicy crayfish. Both cooking techniques involve heating the crayfish for at least 15 min, ensuring the destruction of any potential harmful bacteria or parasites. This not only enhances food safety but also guarantees that the flesh remains tender and the flavors are deeply infused. The way to eat crayfish is to separate the head from the body, and only eat the meat in the tail and the lobster claws (if the liver and pancreas is large enough). The separation of the tail and the head may bring out the yellow liver and pancreas, which some people eat and some people do not eat. All other parts are abandoned.

Most crayfish eaten in China are farmed, although wild varieties are still occasionally enjoyed. The affected patients may have consumed crayfish caught by fishermen following the Yangtze River water flood, sold to the market, and categorized by size during sale (21). Therefore, although most cases involved crayfish purchased from farmers’ markets, we were unable to trace the exact source locations of the crayfish.

Given that we established a sensitive monitoring system to detect the cases of crayfish-related rhabdomyolysis that presented to any hospital since the 2016 outbreak in east China (22), all reported cases were investigated in a timely manner. In this paper, we describe the 423 cases of RM reported from 2016 to 2022, with the majority of them related to crayfish consumption. Although we observed a certain correlation between the number of cases and water levels solely from numerical trends, we still lack robust statistical indicators to conclusively demonstrate this relationship.

Similar to the scenario in Anhui (23), the main clinical manifestation in the 423 cases was muscle pain (most commonly backache followed by the entire body), followed by fatigue. Laboratory results showed that myoglobin and CK levels were the most common abnormal indicators, and most patients demonstrated significant liver function abnormalities without obvious kidney damage (Table 2). These results were consistent with the pathological characteristics of the acute phase of RM, which manifests as striated muscle injury, damage to the cell membrane integrity, leakage of cell contents (such as myoglobin, CK, and small molecule substances), liver dysfunction, and abnormal metabolism (24). In general, there was no obvious renal dysfunction after appropriate treatment.

The disease distribution demonstrated typical regional clustering characteristics. However, the distribution was related to the reported location of the sentinel hospital and the patient’s medical habits. Furthermore, because the crayfish sales in Wuhan are mainly from three large wholesale markets and crayfish is consumed in the entire city through an extremely complex sales chain, the current regional difference in disease incidence does not reflect the true source of pathogenic crayfish.

Furthermore, the study revealed that females and young adults appeared to be more vulnerable to this disease, indicating that they may belong to a high-risk group for Haff disease. However, interestingly, a history of alcohol consumption did not emerge as a significant risk factor for the disease, contradicting some initial hypotheses.

A significant aspect that emerged from these cases was the potential role of excessive crayfish consumption in triggering this disease. It was observed that individuals who consumed a large quantity of crayfish seemed to be at a higher risk of developing this condition. The consumption of crayfish liver and pancreas, consumption location, and crayfish origin were consistent with the results from the suspected foodborne RM outbreak in Anhui and Jiangsu in 2016, which may be related to the crayfish population consumed (7, 8). A significant aspect that emerged from these cases was the potential role of excessive crayfish consumption in triggering this disease. It was observed that individuals who consumed a large quantity of crayfish seemed to be at a higher risk of developing this condition.

Excessive consumption of crayfish (>10) may be a risk factor for the disease. This suggests that the intake of crayfish in large quantities may contain certain components or substances that trigger the onset of rhabdomyolysis.

Braising in sauce, steaming, and braising in oil are three common methods of cooking shrimp, and there is little difference in the cases prepared using these methods. The cooking time for crayfish using these three techniques is also essentially the same (>15 min). It supports the hypothesis that many researchers believe that Haff disease may be caused by a kind of lipid-soluble, heat-stable, and target tissue-specific toxin similar to algal toxin, which is caused by bioaccumulation in the food chain. Crayfish have autoimmunity to this toxin (25). This toxin is not within the current range of known toxin detection, and its properties may be similar to sea anemone toxin and hydra toxin. Cardoso CW hypothesized that the disease was caused by consumption of fish contaminated with palytoxin (PLTX)-like compounds, such as isobaric PLTX, ovatoxin-a (OVTX-a), OVTX-b and OVTX-d (5, 6).

It is plausible that healthy animals were transported to new areas, which introduced pathogens to naive hosts and caused the death of other crayfish within the same area (23). This could be the reason why the incidence of the disease has increased along with the rise in the Yangtze River water level. And why only some people who consume crayfish develop an illness. It is likely that individuals who consume less than 10 crayfish do not develop disease. Importantly, the seasonal increase in crayfish population along the Yangtze River might explain the seasonal outbreaks of Haff disease (24).

Most patients consume crayfish with the intestines removed but without the head and gills, yet the relationship between the consumption of specific parts of crayfish and the onset of the disease remains unclear. Whether it is a particular component in the crayfish, a combination of factors, or something else entirely remains to be discovered. Therefore, further research is imperative to identify the relevant risk factors for this disease and gain a deeper understanding of its etiology and pathogenesis.

Such studies could involve a more detailed analysis of the dietary habits of affected individuals, the biological components of crayfish, and their potential interactions with the human body. Additionally, longitudinal studies could be conducted to monitor the long-term health outcomes of those affected by crayfish-related rhabdomyolysis and identify any potential predictors or moderators of the disease. By gaining a clearer picture of the disease’s etiology and risk factors, we can hope to develop more effective prevention and treatment strategies for those at risk.

The current study faces several limitations. Firstly, it lacks a thorough analysis of risk factors and fails to account for potential confounders, which could have influenced the observed relationships. Secondly, incomplete data exists as some cases have missing interview records. Additionally, toxicological investigations were not conducted, hindering our understanding of the potential toxicological mechanisms involved in the development of RM following crayfish consumption. Future studies should aim to address these limitations and delve deeper into the mechanisms underlying the development of RM after crayfish consumption.

## 5 Conclusion

Crayfish-related rhabdomyolysis stands as an intriguing medical case or cluster of cases, involving patients who present with severe myalgia or weakness of unknown etiology and underlying mechanism. A total of 423 patients were reported to have exhibited symptoms related to this condition in Wuhan, China, 2016–2022, with muscle pain, weakness, and chest distress being the primary complaints. Large crayfish consumption may be a risk factor, while alcohol consumption does not seem to play a significant role. But it remains unclear which crayfish components are associated with the disease’s development. Additional research is required to determine the actual causes of Haff disease.

## Data availability statement

The original contributions presented in the study are included in the article/Supplementary material, further inquiries can be directed to the corresponding author.

## Ethics statement

The studies involving humans were approved by Chinese Center for Disease Control and Prevention Institutional Review Board. The studies were conducted in accordance with the local legislation and institutional requirements. The participants provided their written informed consent to participate in this study.

## Author contributions

YW: Data curation, Writing – original draft, Methodology. XW: Methodology, Writing – review & editing. XYW: Investigation, Resources, Writing – review & editing. ZH: Validation, Writing – review & editing. RW: Visualization, Writing – review & editing. ZC: Writing – review & editing, Software. XMW: Conceptualization, Data curation, Funding acquisition, Project administration, Supervision, Writing – review & editing.
