# Supplementary material for: Surveillance for rhabdomyolysis after the consumption of crayfish in Wuhan, China, 2016–2022
Source: Front Nutr. 2024 May 3;11:1333888. doi: 10.3389/fnut.2024.1333888 (PMC11099252; doi:10.3389/fnut.2024.1333888)
Supplement: Supplementary file 1 [file Data_Sheet_1.PDF]

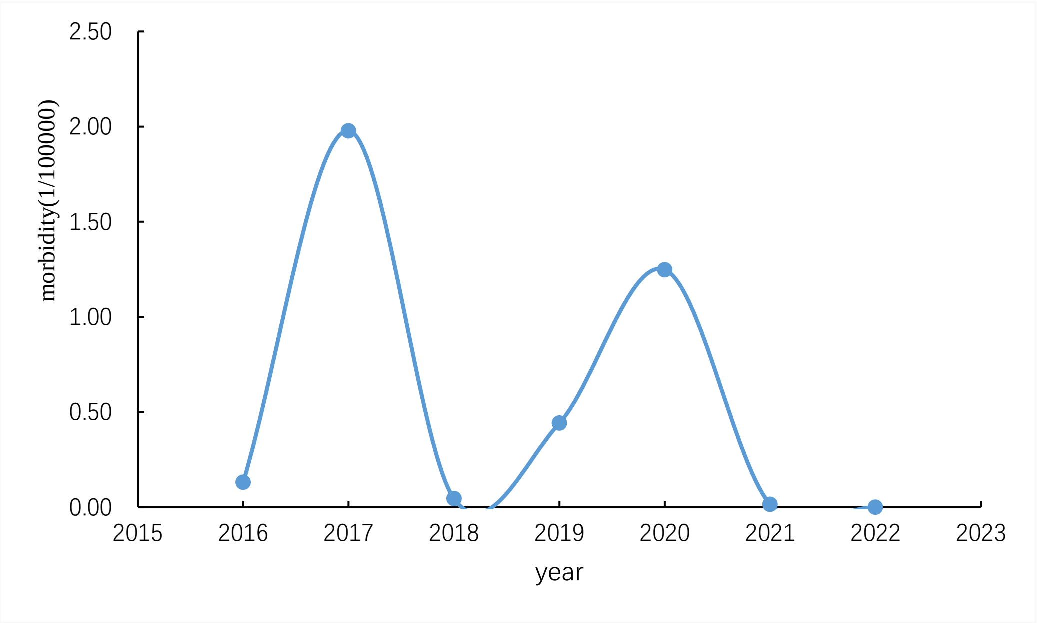

Supplemental Figure 1. The morbidity of rhabdomyolysis during 2016-2022 after the consumption of crayfish in Wuhan, China.

Supplemental Figure 2. Regional distribution of outbreak of Rhabdomyolysis after the consumption of crayfish in Wuhan, China, 2016-2022 (n=423)

regions/year	2016 mobility (1/100000)	2017 Mobility (1/100000)	2018 Mobility (1/100000)	2019 Mobility (1/100000)	2020 Mobility (1/100000)	2021 Mobility (1/100000)	2022 Mobility (1/100000)
Jiang'an	0.00	1.35	0.10	0.00	0.83	0.00	0.00
Jianghan	0.00	10.00	0.27	0.96	1.23	0.00	0.00
Qiaokou	1.39	6.11	0.23	0.92	0.46	0.00	0.00
Hanyang	0.00	2.31	0.00	0.00	0.75	0.12	0.00
Wuchang	0.00	2.03	0.00	0.39	3.58	0.00	0.00
Qingshan	0.00	3.98	0.00	0.00	0.57	0.00	0.00
Hongshan	0.00	0.00	0.00	0.00	0.35	0.00	0.00
Dongxihu	0.00	0.56	0.00	0.00	0.33	0.00	0.00
Hannan	0.00	4.56	0.00	20.62	41.18	0.69	0.00
Caidian	0.28	0.00	0.00	0.13	0.13	0.00	0.00
Huangpi	0.00	0.31	0.00	0.00	0.00	0.00	0.00
